# Exercise and Exercise Mimetics for the Treatment of Musculoskeletal Disorders

**DOI:** 10.1007/s11914-022-00739-6

**Published:** 2022-07-26

**Authors:** Alessia S. Cento, Massimiliano Leigheb, Giuseppina Caretti, Fabio Penna

**Affiliations:** 1 Department of Clinical and Biological Sciences, University of Torino, Corso Raffaello, 30, 10125 Torino, Italy; 2 Orthopaedics and Traumatology Unit, “Maggiore della Carità” Hospital, Department of Health Sciences, University of Piemonte Orientale, Via Solaroli 17, 28100 Novara, Italy; 3 Department of Biosciences, University of Milan, Via Celoria 26, 20133 Milan, Italy

**Keywords:** Exercise, Musculoskeletal disorders, Frailty, Tissue cross-talk, Exercise mimetics

## Abstract

**Purpose of Review:**

The incidence of musculoskeletal disorders affecting bones, joints, and muscles is dramatically increasing in parallel with the increased longevity of the worldwide population, severely impacting on the individual’s quality of life and on the healthcare costs. Inactivity and sedentary lifestyle are nowadays considered the main drivers of age-associated musculoskeletal disorders and exercise may counteract such alterations also in other bone- and muscle-centered disorders. This review aims at clarifying the potential use of exercise training to improve musculoskeletal health.

**Recent Findings:**

Both the skeletal muscle and the bone are involved in a complex crosstalk determining, in part through tissue-specific and inflammatory/immune released factors, the occurrence of musculoskeletal disorders. Exercise is able to modulate the levels of those molecules and several associated molecular pathways.

**Summary:**

Evidence from preclinical and clinical trials supports the adoption of exercise and the future use of exercise mimicking drugs will optimize the care of individuals with musculoskeletal disorders.

## Introduction

During the last decades, the number of old and very old individuals progressively increased, in parallel with an extension of life expectancy, due to health, social and economic improvements. Despite this, increased longevity does not correspond necessarily to prolonged healthspan; indeed, in many cases, a progressive and unavoidable decline of physical function occurs, eventually leading to frailty [1]. For this reason, preserving health and preventing its decline is one of the relevant future societal challenges [2].

Musculoskeletal health (MSH), considered not only as absence of musculoskeletal disorders (MSD) but also as healthy muscles, bones, and tendons that collaborate without pain, is also important for healthy aging, which the World Health Organization described as ‘the process of developing and maintaining the functional ability that enables wellbeing in older age’ [3]. In this context, an active lifestyle can promote the maintenance of musculoskeletal function, possibly preventing the onset of diseases, including insulin resistance, obesity, and cardiovascular disease, allowing individuals to live in good health, maintaining independence and connecting people with the community, thus resulting in economic advantages for the society [4].

MSD can affect bones, joints, and muscles, causing severe long-term pain, stiffness, and loss of mobility. In developed countries and in particular in older adults, chronic pain and disability associated with MSD are the most important factors that influence the quality of life, resulting in frailty, functional decline, and finally in loss of independence [5]. MSD can be divided into major muscle-centered disorders (sarcopenia, frailty, and cachexia) and major bone-centered disorders (osteoporosis and osteoarthritis). The two sides of MSD are strongly interdependent and potentially targeted by integrated interventions, especially those aimed at maintaining an active lifestyle proposing patient-tailored exercise training interventions. Aim of this review is to summarize the pre-clinical and clinical evidence supporting exercise interventions in order to ameliorate MSH.

## Muscle-Centered Disorders

Frailty commonly occurs as a natural consequence of aging and however represents an aberrant condition with an early outbreak and a greater vulnerability, including higher risk for adverse health outcomes such as fractures, hospitalization, and disability, associated with accelerated physical and cognitive decline [5]. Frailty is closely associated with MSD; indeed, musculoskeletal function is an important factor in frailty diagnosis. Recent evidence suggests that incidence of frailty amounts to 15% in people over 65 years old and exceeds 25% in people over 85 years old, with a higher rate in women compared to men [6]. Sarcopenia can be considered an age-related condition, characterized by a consistent reduction of skeletal muscle mass, caused by reduced muscle fiber size, myofiber number and muscle strength, resulting in skeletal muscle degeneration [7]. Skeletal muscle wasting contributes to the development of frailty, although frailty and sarcopenia are clearly delineated conditions [8]. Indeed, modifications of muscle mass are noticed only in 60% of frail people, suggesting that frailty is also the result of impaired muscle function [9]. Muscle wasting can also occur in patients affected by distinct cancer types that develop a complex multifactorial syndrome defined as cancer cachexia (CC) [10]. Indeed, musculoskeletal alterations are known hallmarks of CC, in particular skeletal muscle atrophy and weakness associated with loss of body weight, depletion of adipose tissue, altered metabolism and systemic inflammation [11]. A contributor to CC that is also relevant for frailty and aging sarcopenia is the increase of oxidative stress, a mechanism that stimulates protein breakdown over protein synthesis, increasing ubiquitin proteasome activity, mitochondrial dysfunction and dysregulation of autophagy [12]. A growing number of reports is showing how muscle and bone undergo a bidirectional crosstalk mediated by myokines and osteokines responsible for both sarcopenia and osteopenia in CC [10].

## Bone-Centered Disorders

Osteoporosis is a multifactorial age-related disease that results from genetic and lifestyle factors, characterized by the loss of bone mineral density (BMD) and mass, associated with higher vulnerability fractures and frailty. In physiological conditions, bone maintains its structure regulating the balance between resorption and formation. In aging, changes in hormones and other circulating factors, together with inactivity, move the balance towards bone resorption, impairing bone structure, eventually resulting in osteoporosis and fractures [8]. Despite preclinical results attributed for long to endocrine mechanisms, in particular estrogen and vitamin D deficiency and reduced dietary intake that have a central role in postmenopausal osteoporosis, in the last years the active role played by the immune system has emerged [13]. In this context, several studies focused the attention on the interplay between osteoclasts and immune cells, able to trigger bone destruction in inflammatory diseases, demonstrating the influence of common molecules of both immune and bone systems, including cytokines, chemokines and signaling factors [14]. Similarly, in rheumatoid arthritis (RA), an osteo-autoimmune disease caused by cartilage depletion in inflamed joints, osteoclast stimulation of bone resorption is CD4+ cell-mediated [15]. Consistently, accumulation of T cells in the synovial fluid promotes osteoclastogenesis secreting interleukin-17 (IL-17), which induces the expression of Receptor activator of nuclear factor kappa-Β ligand (RANKL) in synovial fibroblasts and increases local inflammation [16].

## Inter-organ Crosstalk in the Pathogenesis of Musculoskeletal Disorders

MSD can originate from the interaction among several factors, including the inter-tissue crosstalk or intra-tissutal parenchymal / stromal communication. Indeed, muscle cells produce and release myokines, involved in mediating various metabolic, physiological, and immunological effects on organs, including bone, liver, gut, pancreas, adipose tissue, and vessels [17•].

Several studies demonstrated a bi-directional endocrine and paracrine regulation between bones and muscles, in particular increased muscle mass is paralleled by improved BMD, and consequently by decreased fracture risk [18]. The co-existence of osteoporosis and sarcopenia is called osteosarcopenia, a syndrome with overlapping clinical and biological features [19]. The metabolism of muscle and bone is modulated in parallel, in particular amino acid availability can positively influence protein turnover rate in muscle and contribute to bone collagen synthesis. During aging, the musculoskeletal system reduces the utilization of nutrients that regulate cellular proteins and growth factors involved in muscle and bone metabolism, impairing the release of insulin-like growth factor 1 (IGF-1), inhibiting parathyroid hormone and calcium uptake [20]. In addition, hormonal factors, including testosterone and estrogens, are negatively associated with bone loss and muscle atrophy [21]. Upon exercise, muscle contraction stimulates the release of soluble factors, including IGF-1, matrix metalloproteinase-2 (MMP2) and fibroblast growth factor-2 (FGF2), contributing to bone formation and maintenance [22•]. Instead, upon muscle injury, the muscle releases other myokines, including transforming growth factor β (TGF-β) and myostatin, that disrupts bone homeostasis, repair and healing, as confirmed in myostatin-deficient mice, showing higher bone mineral content and density [23]. Among other myokines, irisin is active both in vitro on myoblast cultures, enhancing osteoblast differentiation [24], and in vivo, where mice treated with recombinant irisin showed higher strength and cortical bone mass [25]. In contrast, ciliary neurotrophic factor (CNTF) seems to counteract osteoblast differentiation and bone formation [26]. Although skeletal muscle is considered the biggest endocrine organ in the human body, also bone can exert endocrine functions, secreting from osteoblasts or osteocytes humoral factors, called osteokines [22•]. Among osteokines, osteocalcin can impact on skeletal muscle, as demonstrated by osteocalcin-deficient mice exhibiting decreased muscle mass and by the administration of osteocalcin in wild-type mice resulting in increased muscularity [27].

**Fig. 1:**
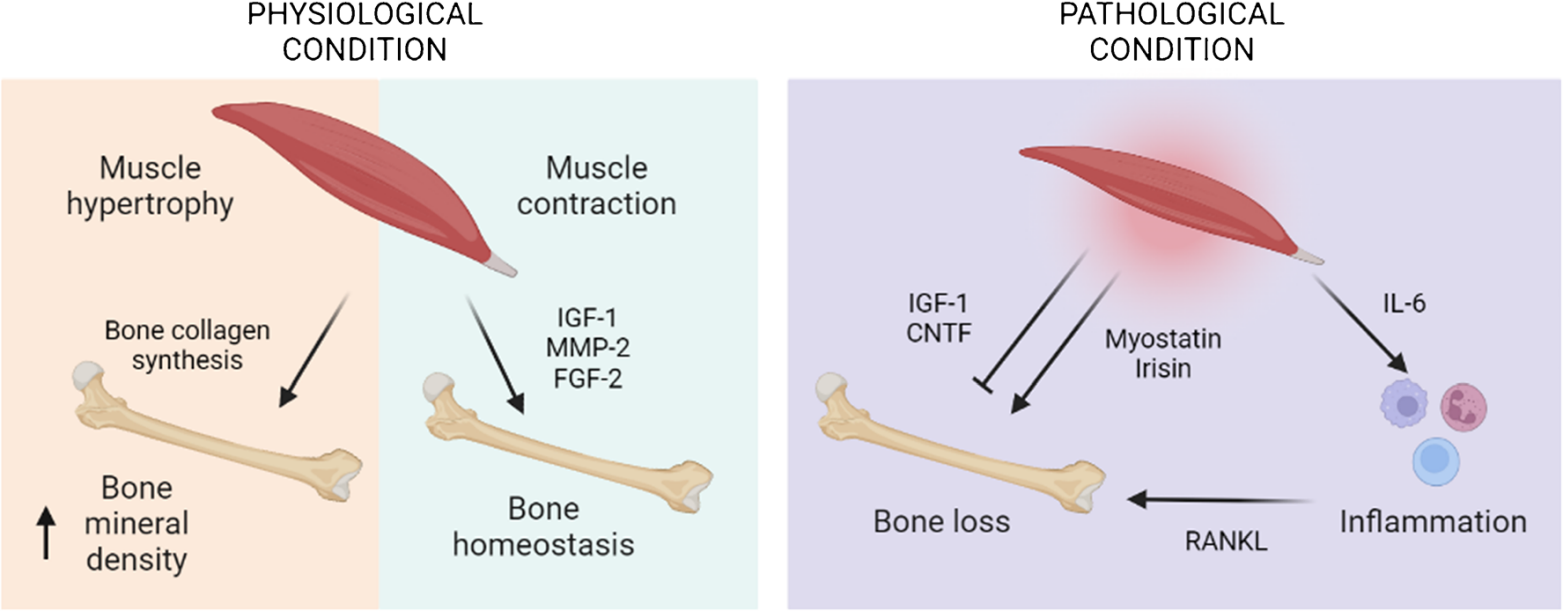
Crosstalk among muscle, bone and the immune system in health and disease. Image created with BioRender.com

Beyond the two main players, muscle and bone, in the last decades the role of muscle in modulating immune function by distinct soluble factors, cell-to-cell interactions and cell surface molecules is being clarified [28]. In particular, myokines including interleukin-6 (IL-6) are able to modulate the immune system and their serum concentrations are negatively associated with age, implicating a correlation between skeletal muscle and immunosenescence, i.e. the loss of immune function occurring during aging [29]. IL-6 can exert anti-inflammatory or pro-inflammatory effects, depending on concentration, duration of exposure and local immune environment, in parallel controlling metabolic processes in muscle, inducing either wasting or hypertrophy [30]. Indeed, IL-6 can promote muscle atrophy and fibrosis, contributing to sarcopenia, by increasing signal transducer and activator of transcription 3 (STAT3) signaling [31] and protein catabolism [32]. Consistently, STAT3 inhibitors administered to dystrophic, aged or injured skeletal muscles promote muscle recovery [33]. Increased levels of IL-6 occur also during muscle wasting in CC [34], and IL-6 inhibition is able to prevent muscle mass loss induced by tumor growth [32]. The other way round, after exercise the skeletal muscle increases IL-6 release depending on the modality and the intensity of exercise, stimulating hypertrophy and satellite cell proliferation in order to restore muscle homeostasis [35]. A non-exhaustive summary of this complex network of interaction in pathophysiology is provided in Figure 1.

## Exercise for Musculoskeletal Disorders: Preclinical and Clinical Evidence

Regular exercise training has positive effects on MSD, ameliorating muscle metabolic homeostasis and inhibiting wasting-associated signaling. In the context of MSD, exercise can be considered as a potential strategy to prevent or at least delay the onset and progression of muscle wasting and sarcopenia on the one side, while minimizing the long-term bone alterations in osteoporosis, osteoarthritis and rheumatoid arthritis (Table 1).

**Table 1:** Selected pre-clinical and clinical studies supporting exercise interventions in musculoskeletal health. The choice of studies was arbitrary and not exhaustive, in order to focus only on the most significant results according to the authors

	Condition	Treatment	Weeks of treatment	Age and sex	Animals/humans	Outcome	References
Muscle-centered diseases	Frailty	Voluntary wheel running	4 weeks	28-30 months M	Mice C57BL/6	Reverse frailty, improve muscle mass and strength	[36]
	Frailty	Voluntary wheel running	13 weeks	21-23 months M-F	Mice C57BL/6	Reverse frailty, increase lifespan in female	[37]
	Frailty	HIIT (10-minute treadmill)	3 times/wk for 4 months	24 months M	Mice C57BL/6	Attenuate frailty, improve muscle and mitochondrial mass	[38]
	Frailty	HIIT (10-minute treadmill)	3 times/wk for 2 months	24 months F	Mice C57BL/6	Attenuate frailty	[39]
	Frailty	Aerobic, resistance, and flexibility exercises	up to 150 min/wk	70-89 years old	Human	Did not attenuate frailty	[42]
	Frailty	Aerobic training	60 min/2 days/wk per 26 weeks	65–85 years old	Human	Attenuate partially frailty	[43]
	Frailty	Aerobic, resistance, and flexibility exercises	60 min/3 days per 16 weeks	≥65 years old	Human	Improve frailty	[44]
	Cachexia	Treadmill running	30 min/5 days/wk per 4 weeks	10-11 weeks F	Mice BALB/c	Ameliorate CC-induced muscle wasting	[47]
	Cachexia	Motorized wheel running	11 m/min for 45 min per 5 days/wk	6 weeks F	Mice BALB/c	Counteract the metabolic impairments	[48]
Bone-centered diseases	Osteoartrithis	Treadmill running	16 m/min, 3 days/wk for 8 weeks	12 weeks M	Wistar rats	Reduce inflammation and improve functional performance	[51]
	Osteoporosis	Treadmill running	6 m/min for 5 min + 8 m/min for 55 min (+1m/min/wk) per 9 weeks	12 weeks F	Mice C57BL/6	Reduce osteopenia	[52]
	Rheumatoid arthritis	Aerobic exercise	Several	44–68 years old	Human	Improve function	[53]

Starting from age-associated frailty and sarcopenia, exercise can improve muscle oxidative capacity, joint flexibility, increasing muscle mass and enhancing muscle strength, finally improving the quality of life [7]. Several studies on pre-clinical models of frailty demonstrated that exercise, aerobic in particular, represents one of the most effective interventions. Graber et al. studied the effect of voluntary wheel running on frailty in mice using a previously established mouse frailty index, demonstrating that 4 weeks of voluntary wheel running improved frailty, muscle mass and strength in 28–30 month-old male C57BL/6 mice compared with 6–8 month-old male mice [36]. Accordingly, Bisset et al*.* demonstrated that a longer time of voluntary wheel running (13 weeks) effectively prevents frailty in both 21–23 month-old male and female C57BL/6 mice compared to sedentary controls, although increasing lifespan only in older females [37]. In parallel, other studies evaluated the potential beneficial effects of high-intensity interval training (HIIT) in attenuating frailty. Seldeen et al. demonstrated that treadmill HIIT sessions for 8–16 weeks attenuated frailty in 22-month-old female and male C57BL/6 mice compared to sedentary mice. HIIT results in increased mitochondrial mass, muscle mass and fiber cross-sectional area only in males [38•, 39]. Also resistance exercise represents an important exercise modality for the treatment of frailty, generating increased amount of type I muscle fibers and inducing type II fiber hypertrophy [40], regulating protein synthesis and autophagy, finally preventing muscle atrophy [41]. Translating such knowledge to humans, despite Trombetti et al. demonstrated that a moderate exercise over a 2-year period did not ameliorate frailty in inactive old individuals [42], other studies showed that exercise can improve frailty in older people, in particular with aerobic training [43] and combining aerobic, muscle strength, flexibility and balance exercises, in order to improve the cardiovascular, muscular and neuromuscular systems [44].

Regular physical activity produces distinct adaptations, such as muscle mitochondrial biogenesis leading to an improved healthspan [45], but also inhibits tumor growth, contributing to spare muscle mass in CC [46]. Several studies measured the effects of exercise on skeletal muscle functional properties in tumor-bearing mice. Morinaga et al. examined the effect of aerobic exercise in CC muscle atrophy, demonstrating that 4 weeks of treadmill running reduces muscle atrophy in C26-bearing mice, without affecting ubiquitin-proteasome system and autophagy [47]. In contrast, Ballarò et al. evaluated the combination of resistance and endurance exercise in chemotherapy-treated C26-bearing mice, confirming previous results on rescue of muscle strength and mass, and demonstrating the prevention of proteolysis induction and the improvement of mitochondrial mass and activity [48]. Despite the recommendations for the management and treatment of CC do not clearly include exercise training due to the lack of clinical evidence [49], exercise currently remains one of the most promising interventions.

While resistance exercise seems to act mainly in the prevention or rescue of muscle mass loss, endurance exercise, that consists in long-duration and low-intensity aerobic exercise, is considered the best option in RA and osteoarthritis (OA) conditions, by improving pain, edema and joint function [50]. Consistently, Martins et al. demonstrated that moderate intensity aerobic training reduces inflammatory marker levels and improves the functional performance of OA animals [51], while Guo et al. evidenced its positive effects in improving bone properties and alleviating osteopenia in mice with osteoporosis [52]. According to these data, a meta-analysis of randomized controlled trials in patients with RA confirmed that aerobic exercise improves function and decreases structural bone damage [53]. 

## Ongoing and Newly Designed Exercise-Based Clinical Trials

As introduced in the previous chapter, the impact of exercise-based interventions has been extensively investigated in human diseased conditions associated with MSD. Considering the two main modalities of exercise, aerobic and resistance training, several meta-analyses considering the studies involving frail people are available. Focusing on the effectiveness of exercise in preventing the risk of falls, an outcome strongly dependent on MSH, both exercise modalities proved to be useful [54], with the better results obtained from mixed interventions. The strongest evidence, however, is for the adoption of progressive resistance training [55], being able to improve BMD, physical function and eventually the quality of life. As for the current clinical research, a clinicaltrial.gov search for the condition ‘osteoporosis’ and the additional term ‘exercise’ retrieved 208 studies, of whom 41 are active, either enrolling or not yet, although part of the studies do not include an exercise-based intervention and only use questionnaires or functional assessments of physical performance. A very huge number of clinical studies is available for arthritis, with 1074 entries found and 301 active studies. On the same line, a lot of information can be obtained from studies including exercise in other chronic conditions associated with problems in MSH such as cancer, renal failure and diabetes, although with a focus that frequently considers bone involvement only marginally. Focusing on the active studies prescribing exercise, most of them target age-related bone loss and the intervention consists of either education to exercise or supervised exercise training, with several exercise modalities.

In the attempt to highlight new trends, there are some studies that are worth mentioning. Some of the studies compare distinct exercise modalities (e.g., ***NCT04815824; NCT05266976***), while others introduce new training activities or combinations (***NCT03885466; NCT02617303; NCT03683849****).* It seems likely that on one side there are studies aimed at defining a ‘gold standard’, required for providing a strong evidence for including exercise in the standard of care for MSD, while, on the other side, the demonstration that many distinct recreational training activities are effective will potentially widen the spectrum of choice for patients having distinct socio cultural interests or simply propensity to perform a given exercise. The latter point is not marginal, since the adherence to exercise prescription is frequently poor [56], for either objective limitations or, more frequently, for subjective variables such as the presence of perceived barriers or the lack of individualization, of social support, of integration with daily life, and others.

Unfortunately, many of the new trials are performed in small cohorts (even less than 100 subjects), are heterogeneous and only minimally comparable. Furthermore, many of those combine nutritional interventions with exercise, making it difficult to discriminate between the effect of exercise from the improvements induced by the diet or not allowing to perform a proper meta-analysis to draw conclusions on exercise effectiveness. The other way round, in the last years, the effort of consortia involved in aging research produced consensus guidelines for the use of exercise as a non-pharmacological intervention [57, 58], defining modalities and doses for a conscious exercise prescription, likely providing the base for new, more standardized, randomized controlled trials.

## Molecular Mechanisms of Exercise-Promoted Musculoskeletal Health

Beyond demonstrating the clinical effectiveness of exercise in the treatment of MSD, it is fundamental to understand the pathways regulated by exercise in order to design in the future mechanism-based interventions. Physical activity can induce negative or positive effects on oxidative stress determined by the intensity and the type of the training. While regular and moderate exercise improves health, stimulating antioxidant defense systems and decreasing the detrimental effect of the peroxidation reactions, intense and stressful exercise induces an overproduction of reactive oxygen species (ROS) and impairment of antioxidant defense system in the skeletal muscles [59]. Indeed, muscle contraction induces ROS production, making it the primary source of ROS during exercise [60••]. As demonstrated by Mota et al., the use of a combined exercise program reduces not only the oxidative stress, but also limits DNA damage and increases antioxidant capacity [61]. A relevant target of exercise training is represented by the nuclear factor erythroid 2–related factor 2 (Nrf2), that is required for exercise adaptations and mediates the defense from skeletal muscle damage induced by ROS [62], as confirmed in Nrf2 knockout mice [63]. Interestingly, mitochondria not only represent a source of oxidative stress, but can themselves be damaged by ROS, resulting in mitochondrial dysfunction. The skeletal muscle adapts to oxidative stress produced during exercise, mainly recruiting the peroxisome proliferator-activated receptor γ coactivator-1α (PGC-1α), a major modulator of mitochondrial function and integrity [64]. Exercise stimulates the expression of PGC-1α in the skeletal muscle, sustaining its crucial role in maintaining mitochondrial metabolic and antioxidant capacity, beyond mitochondrial biogenesis [65]. Similarly, Nrf2 can also exert positive effects on muscle, inducing mitochondrial biogenesis and function [66]. Finally, mitochondrial turnover represents a mechanism responsible for the maintenance of mitochondrial health, indeed, exercise-induced mitophagy can produce beneficial effects on muscle during aging, reducing the accumulation of damaged mitochondria [67]. The mechanisms characterized in mice have been partly confirmed in humans, since moderate intensity exercise increases the mRNA levels of several autophagy related genes such as microtubule-associated proteins 1A/1B light chain 3B (LC3II), autophagy related 12 (Atg12), autophagy related 16 (Atg16) and lysosome-associated membrane protein 2 (LAMP-2) in elderly [68].

Beyond the events occurring within the muscle fiber, it is worth considering that physical inactivity is associated with chronic inflammation as assessed by increased levels of circulating mediators, including C-reactive protein (CRP), tumor necrosis factor α (TNF-α) and IL-6 [69]. In contrast, regular exercise is associated with decreased levels of CRP and IL-6, in parallel to increased levels of the anti-inflammatory factors, such as interleukin-10 (IL-10) and adiponectin [70]. Physical activity can exert its anti-inflammatory effect also reducing monocyte expression of toll-like-receptors (TLRs), as suggested in previously sedentary old individuals undergoing moderate exercise training [71••]. Again, the type and intensity of the exercise can differently modulate the inflammatory response. Although resistance exercise was proposed to be the best approach in order to reduce expression of TNF-α in aged skeletal muscle [72], a more recent study demonstrated that aerobic exercise is more suitable for regulating inflammatory markers and in general the immune system in elderly [73].

## Exercise Mimicking Drugs for the Treatment of Musculoskeletal Disorders

**Fig. 2:**
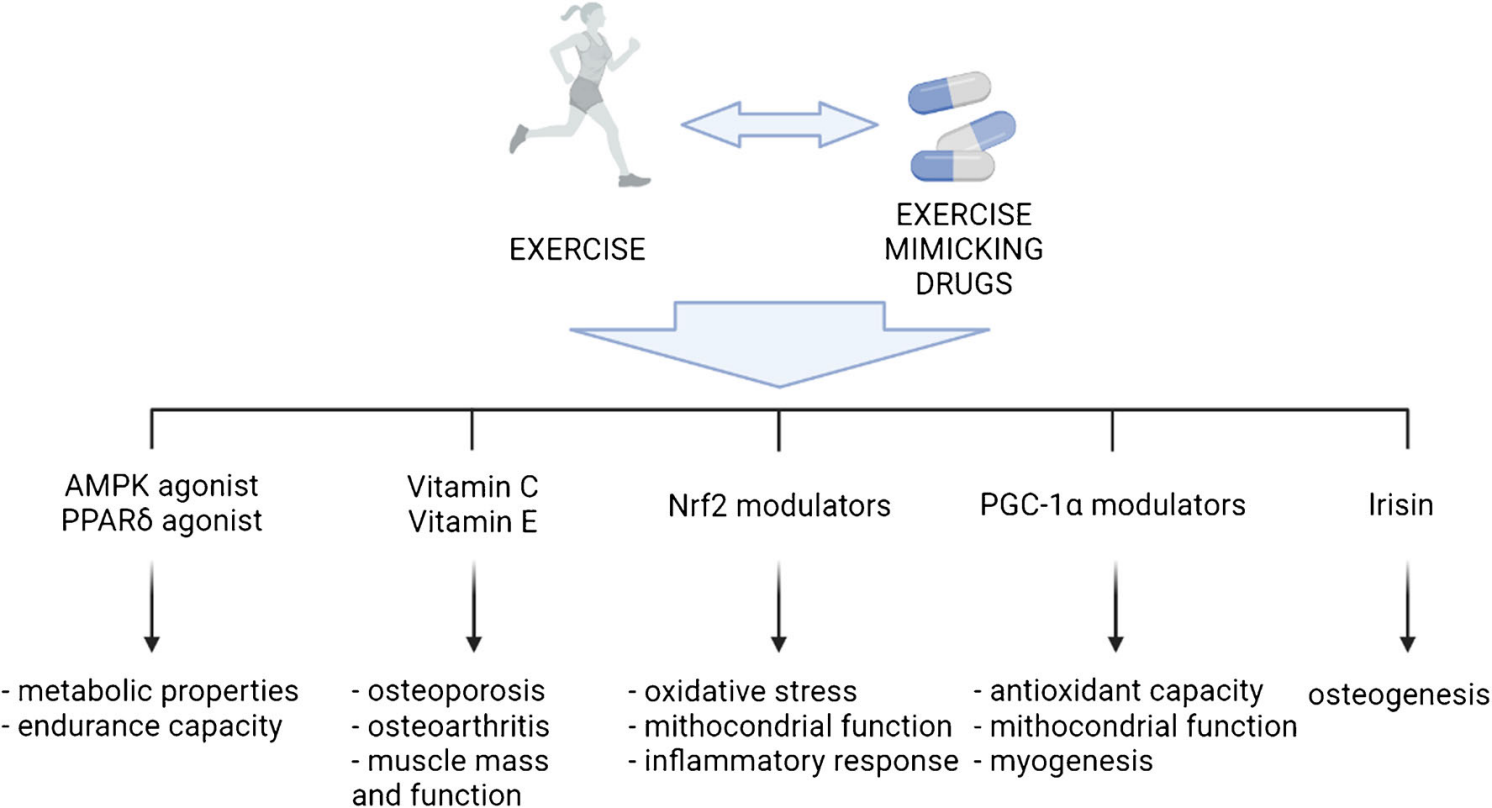
Schematic overview of prospective exercise mimicking drug molecular targets along with the biological process modulated. Image created with BioRender.com

Provided that exercise usefulness for MSH is an indisputable fact, the research community is also investigating the possibility to define pharmacological or nutritional interventions able to mimic and/or to potentiate the beneficial effects of exercise. There are two main reasons for investing in such direction, the first to provide an alternative option to exercise in those individuals that, for any reason, are unable to exercise and, secondly, to widen the therapeutic window and to maximize the exercise-induced improvements, even if the exercise dose is sub-optimal, thus providing a pharmacological ‘exercise pill’. A schematic summary of the main targets of exercise mimetics is presented in Figure 2.

The search for compounds having exercise mimicking properties (exercise mimetics from hereon) started almost 15 years ago with a seminal paper describing the ability of 5' AMP-activated protein kinase (AMPK) and peroxisome proliferator-activated receptor (PPAR-δ) agonists to improve metabolic properties and endurance capacity even in sedentary mice [74]. The majority of the exercise mimetics identified so far have been characterized for their activity on skeletal muscle health and performance, in line with the observation that the favorable action of exercise is mainly dependent on the metabolic adaptation occurring in the skeletal muscle [75] and the consequent cascade induced by the release of metabolites and myokines, improving health and function of other tissues or organs.

Keeping in mind the main molecular pathways modulated by exercise, described in the previous chapter, exercise mimetics impinging on the same mechanisms are available and will potentially impact on MSD. Starting from the modulation of oxidative stress, several compounds behaving as antioxidants have been tested. From the broad list of antioxidants, vitamins such as C and E, frequently present in commercially available nutraceutical supplements, have been proposed for treating both osteoporosis [76] and osteoarthritis [77, 78], simultaneously impacting positively on skeletal muscle mass and function [79]. Given that mitochondria are the main source of ROS, mitochondria-targeted compounds that reduce oxidative stress and improve cellular energy metabolism are desirable. At the crossroads of ROS and oxidative metabolism control, the transcription factor Nrf2 is growing as an ideal target for several age- and chronic disease-associated MSD from both the bone and the muscle perspectives [66, 80]. Several synthetic and natural compounds are able to modulate Nrf2 activity [81], among which many canonical anti-inflammatory drugs already approved for treating MSD. Considering that exercise mimetics reducing oxidative stress and improving mitochondrial function will also produce an anti-inflammatory effect, their use will likely allow to reduce the current excessive use of both steroidal and non-steroidal anti-inflammatory drugs for MSD. Beyond Nrf2, PGC-1α is another nuclear factor fundamental in determining the exercise-induced beneficial effects (see previous chapter). Pharmacological screenings for PGC-1α modulators have long been run and new small molecule PGC-1α activators are now being developed [82], likely providing new therapeutic options also for MSD. Beyond direct PGC-1α activators, metabolic modulators can also impact on PGC-1α improving the oxidative capacity of the skeletal muscle and stimulating the myogenic program in old mice [83]. Our recent observations [84] support the relevance of PGC-1α in promoting the myogenic process in adulthood and in the prevention of the fibro-adipogenic drift frequently observed in the elderly. Linked with PGC-1α, exercise-induced myokines may also be used for treating MSD. Among them, there is large evidence linking irisin with bone health [85]. Indeed, irisin administration was reported to improve osteogenesis and fracture recovery, protecting from dexamethasone-induced cell death and disuse-induced bone loss [86].

Despite the use of exercise mimetics is still far from reaching a generalized consensus, it is worth mentioning a clinical trial (***NCT03227458***) combining exercise with the administration of dehydroepiandrosterone, a natural hormone that potentiates the effects of exercise, in order to maximize the action of exercise in older women, suggesting that the preclinical results are already stimulating the physicians to integrate exercise and exercise mimetics in the clinical setting.

## Conclusions

Recent findings have highlighted the importance of maintaining an active lifestyle that includes structured exercise programs in the prevention of MSD of distinct origin. The interdisciplinary preclinical research merging experts from both the bone and the skeletal muscle fields has allowed the identification of the molecular mechanisms behind MSD and the ‘druggable’ targets that both exercise and new nutritional and pharmacological approaches may hit. On the other side, the number of exercise-based clinical trials against MSD is increasing along with the awareness of the importance of proposing patient-tailored exercise interventions. We foresee a stronger integration of basic with clinical investigators in order to provide mechanism-based interventions that incorporate exercise in multimodal therapies that will effectively improve MSH in the future.

## Declarations

All the figures and table are original and never published before.
